# The Effect of Mindful Leadership on Employee Innovative Behavior: Evidence from the Healthcare Sectors in China

**DOI:** 10.3390/ijerph191912263

**Published:** 2022-09-27

**Authors:** Min Zheng, Zhenting Xu, Yiying Qu

**Affiliations:** 1 School of Logistics, Linyi University, Linyi 276000, China; 2 Business School, Qingdao University of Technology, Qingdao 266525, China; 3 Business School, East China University of Political Science and Law, Shanghai 200042, China

**Keywords:** employee innovative behavior, mindful leadership, creative process engagement, creative self-efficacy

## Abstract

In the health care system, it is increasingly apparent that employee innovative behavior improves the core competitiveness and resilience of organizations. Previous research has identified leadership behavior as a key predictor of employee innovative behavior. Following this logic and by integrating social information processing theory with existing research conclusions, we constructed a moderated mediation model to examine the mechanism by which mindful leadership influences employee innovative behavior. An empirical analysis of 361 questionnaires that were completed by employees from the healthcare sector in China shows that mindful leadership is positively and significantly correlated with employee innovative behavior. Creative process engagement was found to play a mediating role in this relationship. Moreover, creative self-efficacy positively moderated the relationship between mindful leadership and creative process engagement and moderated the mediating effect of creative process engagement on the relationship between mindful leadership and employee innovative behavior. That is, compared with employees with lower creative self-efficacy, employees with higher creative self-efficacy experienced a stronger indirect effect of mindful leadership on their innovative behavior. This study enriches the theoretical research on mindful leadership, clarifies the mechanism and boundary conditions of the effect of mindful leadership on employee innovative behavior, and provides theoretical support for organizational activities that stimulate and guide employee innovative behavior.

## 1. Introduction

In this knowledge-economy era, healthcare sectors exist in a dynamic environment characterized by shortened product life cycles, rapid technological changes, and globalization. To survive, compete, grow, and progress, healthcare sectors must cultivate more innovative behaviors than ever before [1,2]. An example of such behavior is “employee innovative behavior”, which involves employees generating creative ideas and transforming these into useful products or services [3]. This is not typically an in-role behavior that is expected of employees or covered by organizational reward systems; instead, it is an out-of-role behavior that is freely determined by employees [4]. In practice, employees are likely to relinquish innovative behavior due to risk aversion, which is ultimately detrimental to the long-term survival and development of an organization [5,6]. Consequently, effectively stimulating employee innovative behavior is both a practical management problem and an important theoretical area in the field of management.

A growing number of studies have shown that leadership behavior is a key contextual factor affecting employee innovative behavior [7]. Of note, transformational leadership, entrepreneurial leadership, ambidextrous leadership, and authentic leadership all have significant predictive effects on employee innovative behavior [8,9,10,11]. To cope with the challenges of increasing uncertainty and complexity in business environments, leaders must also cultivate keen awareness, an ability to quickly absorb relevant information, and the ability to decompress to ease tension [12]. Consequently, mindful leadership, which emphasizes clear and inclusive awareness and focusing on the present moment, has drawn attention from both business organizations and academia [13]. Mindful leaders manage their subordinates in a focused, aware, and compassionate way, which helps the subordinates to concentrate more on the changing environment and perceive it more accurately than they would otherwise be able to do. Mindful leadership also prompts employees to identify with their leader and experience positive emotions, which in turn encourages them to participate in out-of-role behavior. Therefore, this study will explore the effect of mindful leadership on employee innovative behavior.

Social Information Processing (SIP) theory views leadership behavior as an important source of social cues that enable subordinates to interpret their work environment, with the caveat that individuals’ varying interpretations of the work environment lead to different attitudes and behaviors [14]. Following this logic, mindful leadership advocates acceptance without judgment; that is, avoiding declarations of “good” and “bad” or “right” and “wrong”. This non-judgmental attitude is typically interpreted by employees as tolerance for innovative behavior, and it, therefore, encourages employees to participate in the creative process and ultimately enhances their innovative behavior. Most mindfulness research focuses on the impact of employee mindfulness on employees themselves [15,16], ignoring the possibility that the impact of mindfulness may extend beyond the intra-individual domain into the interpersonal domain, especially in the field of leadership [17]. Given this possibility, this study explored the mediating effect of creative process engagement on the relationship between mindful leadership and employee innovative behavior.

According to SIP theory, individual differences lead to discrepancies in the processing and analysis of social information by cognitive agents [14]. In the cognitive field, self-efficacy also plays an important moderating role in guiding individual behavior [18]. Creative self-efficacy is self-efficacy within the domain of innovation. It is, therefore, important to investigate the influence of context and individual differences, such as in their levels of creative self-efficacy, on their innovative behavior [19]. In this study, we explored the moderating role of creative self-efficacy in the relationship between mindful leadership and creative process engagement. To integrate theoretical and practical concerns, we constructed a model of the effect of mindful leadership on employee innovative behavior.

The findings of this study contribute to the field in three key ways. First, this study enriches our understanding of mindful leadership by revealing the effects of mindful leadership on employee behaviors, as has been called for by previous researchers [13]. Second, from an SIP perspective, this study demonstrates that creative process engagement plays a mediating role in the relationship between mindful leadership and employee innovative behavior. Third, this study illustrates the boundary condition of the relationship between mindful leadership and creative process engagement and enables the construction of a moderated mediation model to delineate the mechanism by which mindful leadership affects employee innovative behavior.

## 2. Theoretical Background and Hypotheses Development

### 2.1. Mindful Leadership and Employee Innovative Behavior

The concept of mindfulness can be traced back to Indian Buddhism. Crucially, mindfulness is not only a state of concentration, impartiality, and clarity with respect to the object of awareness but is also an important practical tool used by Buddhists to approach enlightenment and liberation [20,21]. Kabat-Zinn [22] introduced mindfulness in the clinical field of medicine by using mindfulness stress-reduction (meditation) to relieve patients’ physical and mental pain, emphasizing the conscious attention to the present moment and the non-judgmental cognitive processing of the current physical and mental experience. Since then, mindfulness has been further examined in the fields of psychology and neuroscience and has evolved into a complex concept with multiple meanings relating to mental states, cognitive processes, and abilities [23,24].

In recent years, the field of management has increasingly focused on mindfulness, and especially on mindful behavior by leaders. As such, mindful leadership has emerged as a new and unique leadership style [25]. From a trait perspective, Schuh et al. [26] suggested that mindful leaders pay attention to the moment, perceive the environment keenly, and accept events without judgment. These qualities enable them to generate interpersonal benefits during the management process, especially in terms of the attitudes and behavior of subordinates. From a behavioral perspective, Levey and Levey [27] found that mindful leadership has an impact on both organizational and individual resilience. All of this work suggests that mindful leadership can influence the cognition, attitudes, and behavior of subordinates.

According to SIP theory, leaders have high social status and significant influence within their organizations, are usually regarded as an important source of information, and influence employees’ behavior and attitudes by providing information regarding requirements and work styles [14]. Mindful leaders always focus on what is happening inside and outside the organization; they are not immersed in memories of the past or in future plans or fantasies [28]. This signals to subordinates that they should focus on their current tasks and not distract themselves with other concerns, such as worries about the future. This type of calm organization-wide focus is conducive to creating a climate of mutual trust and tolerance. Most importantly, this open and accepting environment eliminates employees’ concerns about the potential negative consequences of innovative behavior and thus encourages employee innovative behavior.

In addition, mindful leaders are excellent at reflecting on their behavior, which enhances their ability to control their internal environment through attention and emotional regulation and thereby reduce their stress levels [29]. Maruping et al. [30] demonstrated that leaders who are good at managing their own stress can reduce the stress levels of subordinates through emotional contagion. Thus, compared with when a leader’s stress levels are high, when a leader’s stress levels are low, the quality of the relationship between the leader and their subordinates improves and the leader is more likely to provide support to subordinates [31]. Research also showed that, compared with employees who perceive that their leaders do not support them, employees who perceive that their leaders support them are more likely to participate in innovative activities [32], which enhances their innovative behavior. Based on this, we formulated the following hypothesis.

**Hypothesis** **1** **(H1).** *Mindful leadership is positively associated with employee innovative behavior.*

### 2.2. The Mediating Role of Creative Process Engagement

“Creative process engagement” is employee participation in procedures or methods related to creative activities, including problem definition and identification, idea screening and confirmation, and information searching and coding [33,34]. Studies have shown that creative process engagement includes task- or problem-related cognitive skills and motivation processes and interaction with others and has a positive impact on creativity and innovative behavior [33,35].

According to SIP theory, employees’ work environments (e.g., leadership behavior) are an important source of information for employees [14], as they provide cues that help employees to construct and interpret events, and these interpretations then guide employees’ behavior. Mindful leaders can regulate their attention and maintain a high degree of sustained attention to the external environment [28]. These behaviors serve as cues that encourage employees to search for information about the current problem, recode and define the problem, and then solve the problem by selecting creative ideas and developing new solutions.

In addition, mindful leadership involves attention to the present moment and the acceptance of internal and external information without judgment. This sends a positive signal to employees and stimulates their own experiences of the moment. Compared with employees who attend less closely to the present moment, those who attend more closely to the present moment broaden their attentional focus, which helps them to absorb more work-related information and process information more efficiently. Maintaining a broad attentional focus allows employees to access a wide variety of information, such as information on opportunities relevant to their current situation and information that has important implications for the future [36]. Moreover, continual exposure to inconsistent or irrelevant information can help employees to examine and identify current problems, question their existing beliefs and established practices in a task area, and think differently [36], ultimately enhancing their engagement in the creative process. Based on this, we formulated the following hypothesis.

**Hypothesis** **2** **(H2).** *Mindful leadership is positively associated with creative process engagement.*

According to the creativity component model [34,37], creative process engagement has a positive effect on creativity and innovation. Sarooghi et al. [38] found that, compared with employees with lower levels of creative process engagement, employees with higher levels of creative process engagement tended to generate more ideas and suggestions and to focus more on implementing these ideas. Together, these tendencies promote employee innovative behavior. SIP theory suggests that mindful leaders guide their employees to work proactively via unique behavioral cues (e.g., focusing on the present) that encourage them to identify problems, search for information, and generate ideas related to the improvement of organizational products or services. Based on the above literature analysis, we posited that mindful leadership affects employee innovative behavior by influencing employees’ engagement in the creative process. Consequently, we formulated the following hypothesis.

**Hypothesis** **3** **(H3).** *Mindful leadership is indirectly associated with employee innovative behavior via creative process engagement.*

### 2.3. The Moderating Role of Creative Self-Efficacy

Creative self-efficacy, which is an extension of self-efficacy conceptualized by Tierney and Farmer [39], is people’s belief in their ability to complete specific tasks and generate creative results. Creative self-efficacy helps employees to actively cope with the difficulties they encounter during the innovation process, take risks, accept uncertain results, and generate and execute innovative ideas [40].

According to SIP theory, there are individual differences in cognitive agents’ processing and analysis of social information [14]. Given that self-efficacy plays an important moderating role in the cognitive domain, which guides individual behavior, self-efficacy is believed to affect people’s use of cognitive strategies by either enhancing or mitigating the effect of their cognition on their behavior [41,42]. Guided by SIP theory, we speculated that the effect of mindful leadership on employees’ creative process engagement is moderated by creative self-efficacy. Specifically, employees with low creative self-efficacy may have low confidence in their ability to achieve creative outcomes and be reluctant to actively seek out the diverse stimuli and information that stimulate innovation [40]. We predicted that, compared with employees with high creative self-efficacy, those with low creative self-efficacy are less positively affected by mindful leadership, as they continue to choose existing paradigms within their organization instead of reflecting carefully, identifying the current problems, and collecting information to deal with them. This tendency inhibits their creative process engagement. Thus, compared with employees with low creative self-efficacy, employees with high creative self-efficacy will benefit more from mindful leadership. They will take the opportunities offered by their leaders to think deeply about current problems, expand the depth and breadth of the information they collect, and then generate and select creative solutions to problems. This will promote their creative process engagement. Based on this, we formulated the following hypothesis.

**Hypothesis** **4** **(H4).** *Creative self-efficacy moderates the relationship between mindful leadership and creative process engagement, that is, the higher creative self-efficacy, the higher the relationship between mindful leadership and creative process engagement.*

We speculated that creative self-efficacy moderates the effect of mindful leadership on creative process engagement, while creative process engagement mediates the effect of mindful leadership on employee innovative behavior. We posited that creative self-efficacy moderates the mediating effect of creative process engagement on the relationship between mindful leadership and employee innovative behavior. Specifically, we posited that, compared with the employee innovative behavior of employees with lower creative self-efficacy, that of employees with higher creative self-efficacy is more strongly indirectly affected by mindful leadership. We, therefore, arrived at the following hypothesis.

**Hypothesis** **5** **(H5).** *Creative self-efficacy moderates the indirect effect of creative process engagement on the relationship between mindful leadership and employee innovative behavior, such that the higher the creative self-efficacy, the stronger the indirect effect of mindful leadership on employee innovative behavior via creative process engagement.*

**Figure 1:**
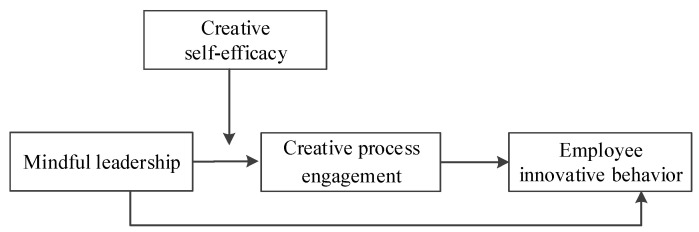
The conceptual model.

Figure 1 represents the conceptual model. In this study, we constructed a moderated mediation model to examine the mechanism by which mindful leadership influences employee innovative behavior.

## 3. Methods

### 3.1. Procedures and Sample

This study used a questionnaire survey to collect the data. The survey respondents worked in Beijing, Shanghai, Qingdao, Jinan, and Chongqing, mainly in biopharmaceuticals, hospitals, clinics, and sanatoriums. To reduce the problem of common method bias, on the one hand, anonymous filling was adopted in this study to ensure that the respondents could fill in the questionnaire with confidence, namely, the respondents were allowed to use abbreviations of their names instead of their real names; on the other hand, 31 alumni network classmates or friends, who were required to assist in the survey, were not permitted to complete the questionnaire in their own department. In addition, this study adopted a longitudinal design by distributing two successive questionnaires at intervals of 3 months. Thus, in January and February 2022, questionnaires were distributed to 605 employees to evaluate mindful leadership and creative process engagement and to collect demographic information. After eliminating incomplete and defective responses, 422 valid questionnaires remained, constituting an effective recovery rate of 69.75%. In April and May 2022, questionnaires were distributed to the 422 employees who had returned usable responses to the first survey. These second questionnaires gathered data on creative self-efficacy and employee innovative behavior. After eliminating the incomplete and defective questionnaires, 361 valid questionnaires were obtained, constituting an effective recovery rate of 59.67%.

The surveyed respondents were predominantly male (52.6% male and 47.4% female). Most of the respondents (70.8%) were aged 25–45, and 20.8% had completed junior college, 54.3% had completed undergraduate education, and 18.3% had completed postgraduate education. Meanwhile, most of the respondents (49.6%) were nurses, 27.3% were doctors, 17.6% were employees from biopharmaceuticals, 3.6% were managers, and 1.9% were others. The majority (78.9%) had been working for either 5–10 years or more than 10 years.

### 3.2. Measures

The instruments we used to measure mindful leadership, creative process engagement, creative self-efficacy, and employee innovative behaviors are all mature and effective scales that have been used previously by scholars. A rigorous translation–back translation procedure was used to translate the instruments into Mandarin. The questionnaire items were measured using a 5-point Likert scale ranging from 1 (“completely disagree”) to 5 (“completely agree”).

Mindful leadership. To measure mindful leadership, according to the measurement method of Schuh et al. [26], which adapted the Mindful Attention Awareness Scale [43] to management and work scenarios, we used 15 items and then selected the 5 items that had the highest loading to measure the level of mindful leadership in an organization. An example item is as follows: “My supervisor has a hard time focusing on what is happening at the moment”. The Cronbach’s alpha coefficient of the scale was 0.918, indicating good reliability.

Creative process engagement. To assess creative process engagement, we adopted the scale developed by Zhang and Bartol [44]. This scale contains a total of 11 items, including “I take enough time to consider the nature of the problem” and “I search for a wider range of information”. The Cronbach’s alpha coefficient of the scale was 0.943, indicating good reliability.

Creative self-efficacy. To measure creative self-efficacy, we adopted the scale developed by Tierney & Farmer [39], which has a total of three items. An example item is as follows: “I am very confident in my creative problem-solving skills”. The Cronbach’s alpha coefficient of the scale was 0.821, indicating good reliability.

Employee innovative behavior. To measure employee innovative behavior, we adopted the scale developed by Scott and Bruce [3], which has a total of six items. An example item is as follows: “I will do everything possible to find new ideas for technologies, processes, and products”. The Cronbach’s alpha coefficient of the scale was 0.904, indicating good reliability.

In line with existing research, we used gender, age, and education as control variables to better clarify the relationship between mindful leadership and employee innovative behavior [45,46].

## 4. Results

### 4.1. Common Method Bias

As the data were self-reported, common method bias was likely to be present, which would have reduced the validity of our findings. Thus, as suggested by Podsakoff et al. [47], we reduced the possibility of common method bias by emphasizing in bold text on the first page of the questionnaire that it was an academic research questionnaire, was not targeted at units or individuals, and that the information we received would be kept confidential. Meanwhile, the participants were also informed that there were no right or wrong answers to the questions; they only needed to choose the option that they thought best described their current situation. Moreover, we conducted the Harman single-factor test of mindful leadership, creative self-efficacy, creative process engagement, and employee innovative behavior. Four factors were extracted, with a cumulative explained total variance of 66.908%, while the maximum variation of factor explanation was 28.333%. This result is in line with the recommended value of the statistical test; that is, it does not exceed 50% of the total variance. We, therefore, concluded that common method bias did not affect the relationship between the target variables.

### 4.2. Confirmatory Factor Analysis

We used IBM SPSS AMOS 23.0 to conduct confirmatory factor analysis (CFA) on four constructs: mindful leadership, creative self-efficacy, creative process engagement, and employee innovative behavior (shown in Table 1). The fit of the hypothetical four-factor model was good, χ^2^/df = 2.577, GFI = 0.909, CFI = 0.932, NFI = 0.924, RMSEA = 0.069, with all of these values meeting the statistical criteria for a good fit [48]. That is, the values of χ^2^/df were 1–5, while those for the CFI, GFI, and NFI were all greater than 0.9, and the RMSEA was less than 0.08. In contrast, the fit for the alternative models we tested was poor. Meanwhile, we conducted the calculation of CR and AVE estimates to further assess convergent and discriminant validity. The results showed that the CR estimates of mindful leadership, creative self-efficacy, creative process engagement, and employee innovative behavior were 0.918, 0.936, 0.804, and 0.892, respectively, which were higher than the recommended value of 0.7. The AVE estimates of mindful leadership, creative self-efficacy, creative process engagement, and employee innovative behavior were 0.693, 0.710, 0.621, and 0.646, respectively, which were higher than the recommended value of 0.5. Based on this, it can be seen that all the constructs had good convergent and discriminant validity.

### 4.3. Descriptive Analysis

The means, standard deviations, and correlation coefficients for each variable are shown in Table 2. As can be seen, mindful leadership was positively and significantly correlated with creative process engagement (r = 0.47, *p* < 0.01) and employee innovative behavior (r = 0.45, *p* < 0.01), and creative process engagement was positively and significantly correlated with employee innovative behavior (r = 0.68, *p* < 0.01). These results were consistent with our expectations and provided a strong analytical premise for subsequent hypothesis testing.

### 4.4. Hypothesis Testing

**Main effect.** We used SPSS Macro Process to test the hypotheses. The results of the regression analyses are shown in Table 3. Model 2, for example, demonstrated that mindful leadership had a significant positive effect on employee innovative behavior (β = 0.33, *p* < 0.001). H1 was therefore supported.

**Mediating test.** Guided by the method of Baron and Kenn [49], we used SPSS Macro Process (model 4) to test the mediating effect. Model 1 showed that mindful leadership had a positive and significant effect on creative process engagement (β = 0.67, *p* < 0.001). H2 was therefore supported. Model 2 added the mediating variable (creative process engagement) and independent variable (mindful leadership) to test the mediating role of creative process engagement. We used the bootstrap method of Preacher et al. [50] with random sampling times set to 5000. The value of the mediating effect of creative process engagement was found to be 0.2280, the 95% confidence interval was [0.1662, 0.2932], and it did not include 0, indicating that mindful leadership had a significant indirect effect on employee innovative behavior via creative process engagement. H3 was therefore supported.

**Moderating effect.** In this study, SPSS Macro Process (model 7) was used to examine the moderating effect of creative self-efficacy (shown in Table 4). Model 3 showed that the interaction between mindful leadership and creative self-efficacy had a significant effect on creative process engagement (β = 0.06, *p* < 0.001), indicating that creative self-efficacy played a moderating role in the relationship between mindful leadership and creative process engagement. H4 was therefore supported.

**Figure 2:**
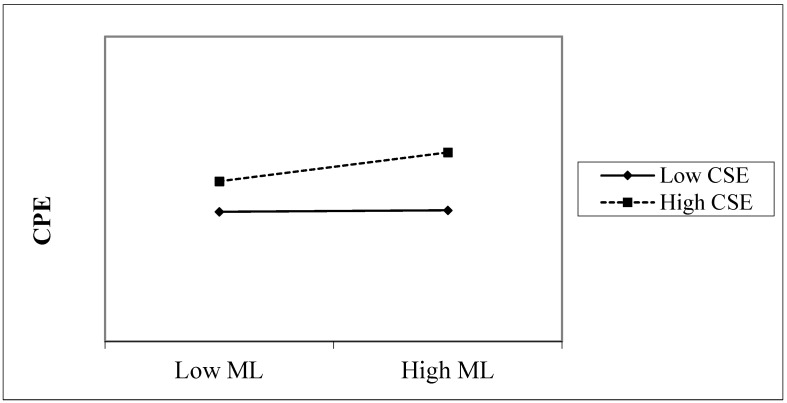
The interaction effect of CSE on the relationship between ML and CPE.

Using the simple slope method recommended by Aiken and West [51], we created an interactive effect diagram (Figure 2) to show the effect of the interaction of mindful leadership and creative self-efficacy on creative process engagement. As can be seen in Figure 2, when creative self-efficacy was low, the relationship between mindful leadership and creative process engagement was nonsignificant (β = 0.30, *p* > 0.05). However, when creative self-efficacy was high, the relationship between mindful leadership and creative process engagement was significant (β = 0.37, *p* < 0.01). These results are consistent with H4.

**Moderated mediation.** The SPSS macro process was used to test the moderated mediating effect. The mean value of the moderating variable was divided into three groups, with one standard deviation plus or minus, and the mediating effect of creative process engagement at different levels of creative self-efficacy was compared. The results are shown in Table 4. For low and high creative self-efficacy, the mediating effect of creative process engagement was significant, respectively, (95% confidence interval [0.0322, 0.1523] and [0.0688, 0.1575]), and the influence coefficient increased from 0.0948 to 0.1139. The 95% confidence interval of creative process engagement was [0.0162, 0.0791], which indicated that the higher the creative self-efficacy was, the stronger the indirect effect of mindful leadership on employee innovative behavior via creative process engagement was, that is, H5 was supported.

## 5. Conclusions and Discussions

### 5.1. Conclusions

Based on SIP theory, this study explored the mechanism by which mindful leadership influences employee innovative behavior, focusing on the mediating role of creative process engagement and the moderating role of creative self-efficacy. The results showed that mindful leadership had a positive and significant effect on employee innovative behavior. Specifically, mindful leadership not only positively affected creative process engagement but also significantly affected employee innovative behavior through creative process engagement. In addition, creative self-efficacy positively moderated the relationship between mindful leadership and creative process engagement and the indirect effect of creative process engagement on the relationship between mindful leadership and employee innovative behavior.

### 5.2. Theoretical Implications

By integrating SIP theory and previous research findings, this study explored the relationship between mindful leadership and employee innovative behavior to answer the questions of “whether”, “how”, and “when” mindful leadership affects employee innovative behavior.

Our work makes three main theoretical contributions. First, researchers who study the relationship between leadership and innovative behavior generally believe that leaders, as an important situational influence and driving force, influence employee innovative behavior by affecting their employees’ cognition and motivation. However, most research has focused on the effects of transformational leadership, entrepreneurial leadership, ambidextrous leadership, and authentic leadership, and has ignored the relationship between mindful leadership and employee innovative behavior. Therefore, this study tentatively explores the relationship between mindful leadership and employee innovative behavior. The results of this study not only concur with previous research that has found that leadership behavior is an important predictor of employee innovative behavior but also expand the field, as we assessed mindful leadership. This enriches our understanding of factors that influence employee innovative behavior.

Second, SIP theory holds that leadership behavior, as an important source of information, provides subordinates with social cues that they use to interpret the work environment. Individuals’ different interpretations of their work environment lead to different attitudes and behaviors [14]. We examined the cognitive processes by which mindful leadership affects employee innovative behavior from the perspective of creative process engagement, which showed that mindful leadership indirectly affects employee innovative behavior via employees’ creative process engagement. These findings helped us to open the “black box” of the influence process of mindful leadership on employee innovative behavior, providing an important theoretical perspective for explaining the mechanisms by which leadership behavior affected employee innovative behavior.

Finally, based on SIP theory, we explored creative self-efficacy (a form of individual difference) by examining its moderating effect on the relationship between mindful leadership and creative process engagement and on the indirect effect of creative process engagement. Our findings reveal and clarify the boundary conditions of the effect of mindful leadership on employee innovative behavior and enrich our understanding of the contextual characteristics of employee innovative behavior.

### 5.3. Practical Implications

Employee innovative behavior is particularly important for organizational effectiveness and survival. How to stimulate employee innovative behavior has become an important and challenging question for leaders. In this study, we found that mindful leadership has a positive effect on employee innovative behavior. This suggests that the leaders of healthcare sectors would benefit from mindfulness training interventions, such as regular or occasional participation in activities involving mindfulness and meditation training and body scans. These activities would improve the mindfulness skills of leaders and thus enhance the ability of their employees to self-regulate, thereby increasing innovative behavior across the organization.

Our results also suggest that to promote employee innovative behavior, in the healthcare sector, leaders should provide employees with sufficient resources (e.g., training projects, resource investment, and time) to allow them to engage fully with the creative process. For example, employees could be scaffolded to participate in problem identification and information searches, which would help them to engage in the process and ultimately promote innovative behavior.

Our finding that creative self-efficacy has a positive and significant moderating effect on the relationship between mindful leadership and employee innovative behavior suggests that organizational managers in the healthcare sector can stimulate employee innovative behavior by cultivating employees’ creative self-efficacy. Activities to increase creative self-efficacy could include daily reviews of work achievements, the development of surrogate role models, instruction on verbal persuasion, creative role identification, and arousal-based methods.

### 5.4. Limitations and Future Research Directions

This study was a preliminary exploration and thus has several limitations. First, we used a cognitive perspective and SIP theory as the basis for our exploration of the mechanism by which mindful leadership affects employee innovative behavior. However, this study neglected other perspectives (e.g., leader–member exchange, positive affect) to explore the mechanism by which mindful leadership affects employee innovative behavior. Therefore, in the future, this mechanism could be examined from other perspectives, such as social exchange, emotional-event, and social learning theory perspectives.

Second, we used a dual-time-point method to collect data. Thus, although common method bias was reduced to some extent, the causal relationship between mindful leadership and employee innovative behavior is not robust. Thus, longitudinal sequential studies are required in the future. For example, data could be collected at multiple time points and longitudinal analysis used to explore in detail the causal relationship between mindful leadership and employee innovative behavior.

Finally, the data for this study (including the evaluations of mindful leadership) were collected directly from employees, which led to measurement bias. In the future, self-evaluations and evaluations by others could be paired to further enrich the data.

## Figures and Tables

**Table 1:** Confirmatory Factor Analysis.

Model	χ^2^/df(df)	GFI	CFI	NFI	RMSEA
Model 1: ML; CSE; CPE; EIB	2.577(269)	0.909	0.932	0.924	0.069
Model 2: ML; CSE + CPE; EIB	4.027(272)	0.781	0.885	0.854	0.092
Model 3: ML; CSE; EIB + CPE	4.566(272)	0.747	0.865	0.834	0.100
Model 4: ML; CSE + EIB + CPE	6.121(274)	0.697	0.755	0.730	0.103
Model 5: ML + CSE + CPE + EIB	8.255(275)	0.598	0.723	0.698	0.142

Note: ML denotes mindful leadership; CSE denotes creative self-efficacy; EIB denotes employee innovative behavior; CPE denotes creative process engagement; “+” denotes the combination of variables.

**Table 2:** Means, standard deviations, and correlations.

Variables	1	2	3	4	5	6	7
1. Gender							
2. Age	−0.12						
3. Educational level	0.08	0.10					
4. Mindful leadership	−0.07	−0.14	−0.05				
5. Creative self-efficacy	0.02	−0.08	0.01	0.38 **			
6. Creative process engagement	−0.07	−0.07	0.03	0.47 **	0.74 **		
7. Employee innovative behavior	−0.06	−0.02	0.02	0.45 **	0.69 **	0.68 **	
Mean	---	3.55	2.84	3.30	3.93	3.95	3.94
Standard deviations	---	1.47	0.79	0.79	0.58	0.51	0.53

Notes: ** *p* < 0.01; N = 361; gender = “male” (1), “female” (2); age = below 25 years old (1), 25 to 35 years old (2), 36 to 45 years old (3), over 45 years old (4); Educational level = junior college (1), undergraduate (2), postgraduate (3), PhD (4).

**Table 3:** Hierarchical regression results of main effect and mediating effect.

Dependent Variables	Creative Process Engagement (M1)			Employee Innovative Behavior (M2)		
Measures	β(SE)	t	*p*	β(SE)	t	*p*
Gender	−0.07(0.05)	0.87	0.387	0.01 (0.04)	1.168	0.284
Age	−0.01(0.02)	−0.47	0.637	0.02 (0.01)	1.335	0.183
Educational level	0.02(0.03)	1.048	0.295	−0.01 (0.02)	0.331	0.741
Mindful leadership	0.67(0.03)	7.34	0.000	0.33 (0.03)	5.593	0.000
Creative process engagement				0.63 (0.04)	15.968	0.000
Index of Mediation	index (SE)		LLCI		ULCI	
0.2280	0.03		0.1662		0.2932	

Note: N = 361.

**Table 4:** Hierarchical regression results of the moderating and moderated mediation effect.

Dependent Variables	Creative Process Engagement (M3)		
Measures	β (SE)	t	* p*
Gender	−0.06 (0.05)	0.71	0.480
Age	−0.01 (0.02)	0.55	0.582
Educational level	0.03 (0.03)	0.86	0.390
Mindful leadership	0.10 (0.03)	3.30	0.001
creative self-efficacy	0.29 (0.04)	4.09	0.000
Mindful leadership * creative self-efficacy	0.06 (0.03)	3.57	0.000
Index of moderated Mediation	Index (SE)	LLCI	ULCI
0.0948 (Low CSE)	0.03	0.0322	0.1523
0.1075 (Medium CSE)	0.02	0.0657	0.1489
0.1139 (High CSE)	0.02	0.0688	0.1575
0.0191 (Diff CSE)	0.01	0.0162	0.0791

Note. N = 361; * denotes the interaction; CES denotes creative process engagement.

## Data Availability

The data that support the findings of this study are available from the authors upon reasonable request.
